# Effect of Heat Treatment on the Microstructure and Mechanical Properties of Rotary Friction Welded AA7075 and AA5083 Dissimilar Joint

**DOI:** 10.3390/ma16062464

**Published:** 2023-03-20

**Authors:** Aditya M. Mahajan, Nagumothu Kishore Babu, Mahesh Kumar Talari, Ateekh Ur Rehman, Prakash Srirangam

**Affiliations:** 1 Department of Metallurgical and Materials Engineering, National Institute of Technology, Warangal 506004, India; 2 Department of Industrial Engineering, College of Engineering, King Saud University, Riyadh 11451, Saudi Arabia; 3 Warwick Manufacturing Group, University of Warwick, Coventry CV4 7AL, UK

**Keywords:** rotary friction welding, aluminum alloys, AA7075 T6511, AA5083 H116, microstructure, hardness, mechanical properties

## Abstract

The present work aims to investigate the changes in the microstructural and mechanical properties of various pre- and post weld heat treatments (PWHTs) on rotary friction welded dissimilar (AA7075 and AA5083) aluminum alloys. The investigation focused on the evolution of weld macro- and microstructures, as well as the changes in hardness and tensile properties resulting from friction welding. The joint integrity was studied through various characterization techniques, and no cracks or incomplete bonding was observed. The study found that the dissimilar joints of the AA7075 and AA5083 alloys displayed higher flash formation on the AA7075 side, which has a lower melting temperature compared to the AA5083 alloy. Various zones were identified in the weld region, including the dynamic recrystallized zone (DRZ), the thermomechanically affected zone (TMAZ) consisting of TMAZ-1 (elongated grains) and TMAZ-2 (compressed/distorted grains), the heat-affected zone (HAZ), and the base metal (BM) zone. The rotary friction welded sample AA5083/AA7075-PWHT joint exhibited the highest strength (yield strength (YS): 195 ± 3 MPa, ultimate tensile strength (UTS): 387 ± 2 MPa) among all the other welded conditions, and this may be attributed to the major strengthening precipitates MgZn_2_ (of AA7075) formed during postweld aging. All dissimilar welds failed in the HAZ region of the AA5083 side due to the formation of coarse grains, indicating the weakest region.

## 1. Introduction

Aluminum alloys are widely used in automotive, aerospace, and construction applications due to their high strength-to-weight ratio, low density, and excellent mechanical and corrosion properties. Among the commercial aluminum alloys available, 7xxx series aluminum alloys possess the highest strength and are used in applications such as heavy aircraft forgings, armor plates, military bridging, heavy goods vehicles, and high-strength structural members. The main alloying element of this alloy, i.e., zinc, has the highest solid solubility in aluminum and generally improves precipitation hardening properties [1]. Additionally, 5xxx series alloys are used in cladding, vessels, tanks, vehicles, vessel hulls, and superstructures [2]. Magnesium is the primary alloying element in 5xxx alloys, which are strengthened by strain hardening.

Cevik [1] has studied the effect of gas tungsten arc (GTA) welding on microstructural properties of 7075 aluminium alloy. He observed that higher thermal exposure led to the formation of large and coaxial grains in the weld nugget. Moreover, hot cracks were detected in the welded samples. The solidifying grains during the fusion welding grew in the direction opposite to the thermal gradient of the weld pool, resulting in columnar grains in the solidified weldment. Further, the solute rejection and microsegregation of the elements during the weld solidification caused the accumulation of low-melting-point liquid at the interdendritic spaces at the terminal stages of the solidification. This, combined with the stresses generated because of the solidification shrinkage, resulted in cracks in the solidifying weldment. The impact strength was negatively affected due to microcracks, heat input, grain size, and hot cracking. Additionally, melting and solidification during the fusion welding resulted in brittle interdendritic structures, which adversely affected the mechanical properties of the joints [3].

During fusion welding of strain-hardened aluminum alloys (such as the 5xxx series), higher heat input causes loss of cold work, which reduces mechanical properties and weld distortion [4]. In the joining of dissimilar metals, mutual solubility plays a vital role along with other factors, such as thermal expansion. The mixing of the two dissimilar alloys often results in the formation of unwanted phases and intermetallic and weld solidification-related issues [5]. The joint may fail due to thermal stresses either during solidification or afterward. This is due to internal stresses present in the brittle intermetallic zone of the weldment [6]. Further, the susceptibility of aluminum alloys to solidification cracking is highly dependent on the weld metal composition, which is influenced by the filler metal and base metal composition, as well as the degree of dilution [7]. Therefore, it is crucial to carefully select the filler metal composition and welding parameters to ensure that the resulting weld composition is not prone to solidification cracking. While this is typically achievable in fusion welding of similar aluminum alloys, selecting the appropriate filler metals for dissimilar aluminum alloy combinations is a challenging task. In fact, for many dissimilar aluminum alloy combinations, there are no filler metals available that can produce welds free of cracks [8]. Even when a suitable filler metal is available, it may not be possible to attain satisfactory joint efficiencies. Due to these challenges, the industry generally avoids fusion welding of dissimilar aluminum alloys. It is evident that successful welding of dissimilar materials is a strenuous task due to various factors, such as the physical properties (such as different melting points and coefficient of thermal expansion) of the base metals [5].

Solid-state welding is a more suitable alternative technique to fusion welding where weldment does not suffer from fusion and subsequent solidification-related issues, as well as the formation of unwanted phases and microstructures [9]. Various types of solid-state welding are available, such as friction stir welding (FSW), cold welding (CW), diffusion welding (DFW), etc. Rotary friction welding (RFW) is a promising technique, as it provides tailored joints based on the required application, short cycle time, less energy loss, and no consumables required. In this technique, joints are produced with the help of heat generated by mechanical friction between the contacting surfaces and pressure. Li et al. [10] studied the effect of rotational speed on the mechanical properties of rotary friction welded AA6061-T6 alloys and observed that rotary speed plays a vital role in developing microhardness and tensile strength. They found that the optimum speed for rotary friction welding of AA6061 T6 alloys was 900 rpm, achieving an overall 88% joint efficiency with the highest strength of 259 MPa.

Ashfaq and Rao [11] compared the bond formation mechanism of rotary friction welded similar (AA2024 and AA6061) and dissimilar (AA2024/AA6061) aluminum alloys. They reported that the burn-off length had no significance on the rotary friction welded similar aluminum alloys. However, in the case of dissimilar welds, the burn-off length had a commensurate impact on strength. Srinivasa et al. [12] analyzed the microstructure and the mechanical properties of friction stir welded AA7075-T6511 aluminum alloys. They obtained high-quality, defect-free welds. However, a notable loss of hardness occurred in the HAZ of either side, resulting from higher heat inputs during welding. The thickness of the plates had a negligible effect on the tensile properties of the weld interface. Further, TEM analysis revealed the absence of precipitates along the grain boundaries in HAZ was responsible for the lower strength of the weldment. Meng et al. [13] describe various approaches that have been developed to optimize friction stir welding parameters, such as rotational speed, traverse speed, and tool geometry. Techniques that have been developed to mitigate defects, such as voids, cracks, and cavities, are also described, including tool design optimization, postweld heat treatment, and the use of filler materials. They also provide a comprehensive overview of the current control strategies that have been developed to address the inherent issues in FSW [13].

Elumalai et al. [14] studied the effect of various welding parameters, such as rotational speed, friction pressure, and forging pressure on the rotary friction welded joint (AA7068/AA7068). They concluded that rotational speed produced the maximum effect on the weld strength followed by friction pressure and forging pressure. Chainarong et al. [15] successfully joined SSM356 and SSM6061 aluminum alloys produced by the gas induced semisolid casting technique (GISS) through rotary friction welding. They reported greater strength and microhardness within the weld interface and thermomechanical zone (TMAZ), which was caused due to higher rotary speed and burn-off length. Fukumoto et al. [16] investigated the amorphization of dissimilar rotary friction welded AA5052 and SS304. They found that larger grains formed along the periphery of the weldment than at the center of the weld zone (dynamically recrystallized zone). Subsequently, greater hardness was obtained at the center (axial) region of the weld interface than at the circumference.

Kumar et al. [17] investigated the effect on the microstructure, mechanical, and corrosion behavior of postweld heat treatment in friction stir welded AA7075 aluminum alloys. They reported lower hardness in the weld nugget due to the dissolution of less stable phases (GP and η′). However, an increase in hardness was seen in TMAZ due to the formation of and growth of η′(MgZn_2_) phases during retrogression and reaging (RRA) heat treatment. Coarser grains were observed in the T6 condition compared to the RRA condition. Further, in RRA samples, corrosion susceptibility was found to be reduced due to the discontinuous grain boundary precipitates with large spacing and a lack of continuous chain for corrosion. On the other hand, T6 samples with finer grains and continuous precipitates resulted in pitting corrosion. Priya et al. [18] examined the effect of postweld heat treatment (PWHT) on the mechanical and microstructural properties of friction stir welded AA6061/AA2219. The lowest hardness was observed in the heat-affected zone (HAZ) of 6061 alloys in the as-welded condition. A significant increase in the hardness was observed after the PWHT (artificial aging) at 165 °C for 18 h; however, there was no effective enhancement in the hardness of HAZ. A postweld solution treatment at 520 °C followed by aging at 165 °C for 18 h resulted in a considerable increase of hardness across the entire weldment. This improvement was also observed in the tensile strength of the joint.

Sasmito et al. [19] investigated the static and fatigue properties of rotary friction welded AA7075/AA5083 at various rotational speeds. They reported that low rotational speed (370 rpm) was insufficient to generate the heat required for bond formation. Moreover, excess rotational speed (2500 rpm) lead to a high burn-off length and wastage of material. The best quality welds were obtained at 1200 rpm. Reprecipitation during welding led to higher hardness at the TMAZ zone of the weld interface. The results showed that increasing the rotational speed of the welding process resulted in a decrease in the tensile strength of the joints. However, the joints welded at higher rotational speeds exhibited higher fatigue strength than those welded at lower speeds. This phenomenon was attributed to the formation of a fine and homogeneous microstructure with a reduced intermetallic compound layer thickness at higher rotational speeds.

To the authors’ knowledge, very limited data is available on the microstructural and mechanical properties of rotary friction welded dissimilar aluminum alloys. The present study is aimed to study the effect of heat treatments on the microstructure and mechanical properties of rotary friction welded AA7075 T6511 to AA5083 H116 aluminum alloys in various heat treatment conditions.

## 2. Experimental Procedure

### 2.1. Base Metal

Base metal AA7075 T6511 solution-treated and aged (STA), AA7075 solution-treated (ST), and AA5083 H116 rods of length 100 mm and diameter 20 mm were used for continuous rotary friction welding. Table 1 shows the chemical composition of the base metals used for the study.

### 2.2. Heat Treatments

To study the influence of pre- and postweld heat treatments on the microstructural and mechanical properties of welded samples, three heat treatment conditions were employed. The AA7075 base samples with two different preweld heat treatments, viz., ST and STA conditions, were used in this study for welding. The solution-treated AA7075 samples were joined via rotary friction welding to AA5083, denoted as AA5083/AA7075-ST. AA7075 (base) solution-treated samples were artificially aged at 120 °C for 24 h and welded to AA5083, denoted as AA5083/AA7075-STA. The welded samples AA5083/AA7075-ST were subjected to postweld aging (PWHT) at 120 °C for 24 h to improve the mechanical properties of the welded sample by precipitate hardening of AA7075, denoted as AA5083/AA7075-PWHT. Table 2 shows the details of these heat treatments.

### 2.3. Welding Details

**Figure 1:**
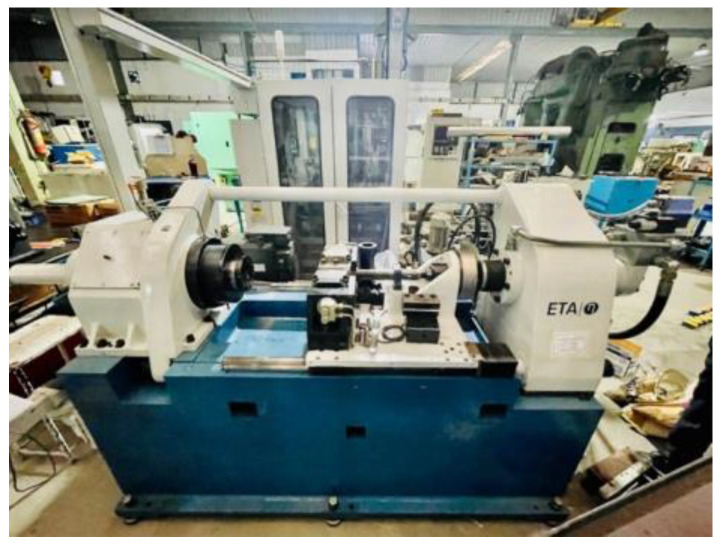
Rotary friction welding setup.

Welding was achieved by a 100 KN capacity continuous drive rotary friction welding machine at ETA Technology, Bangalore, India. The dissimilar (7075 and 5083) base samples were fixed to the rotating chuck and stationary clamp and brought together in contact with an initial force known as frictional pressure. As the spindle started to rotate (spindle speed), the outer oxide surface was broken, and the material began to soften locally due to frictional heat produced during welding. Plastic deformation initiated as it continued to rotate, and more heat generated along the weld interface. An outward plastic metal flow carried the shattered oxide layers (known as a flash) away from the weld until the nascent metals were in contact. After sufficient plastic deformation, the moving parts abruptly came to rest, and a greater axial pressure (upset pressure) was applied to end the welding process [20]. Figure 1 displays the rotary friction welding machine used to weld the metals. An industrial-grade personal computer displayed controlled and stored data, such as rotational speed, soft force, burn-off, etc. The welding parameters used are given in Table 3.

### 2.4. Metallographic Examination

To examine the weld zone microstructure, the samples were lacerated along the cross-section using wire-cut electrical discharge machining (EDM) and then cold-mounted. Samples were then mechanically polished using 600 to 2000 grit SiC papers along with fine cloth polishing by 3–0.5 μm diamond paste. Keller’s reagent (1 mL hydrofluoric acid, 1.5 mL hydrochloric acid, 2.5 mL nitric acid, 95 mL parts water) was employed for etching around a time period of 15–20 s. An optical microscope and a stereomicroscope were used to examine the macro- and microstructural analysis. A microstructural study across the weld interface was carried out by scanning electron microscope (TESCAN, Model: VEGA 3 LMU, Brno, Czech Republic) to obtain a better revolution. The parameters for this examination were 20 kV accelerating voltage with a working distance of 9.38 mm. Energy dispersive X-ray (EDS) analysis was employed to investigate the elemental composition across the weldment.

### 2.5. Mechanical Testing

**Figure 2:**
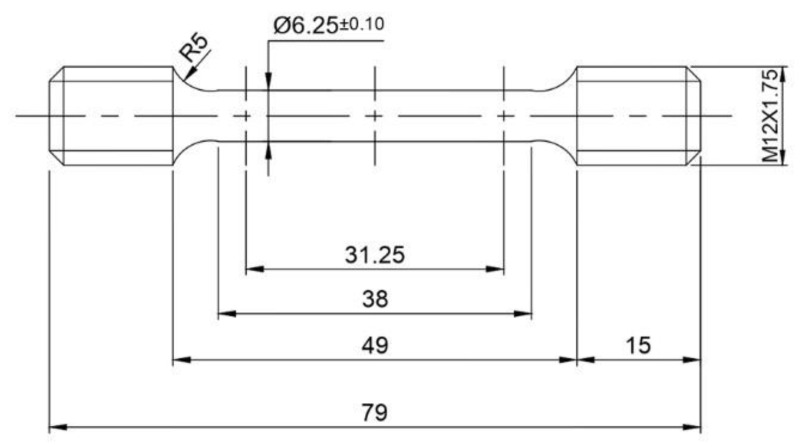
Tensile sample prepared as per ASTM standards.

Mechanical properties, such as microhardness, tensile strength, and fracture properties, were studied in both as-welded and PWHT conditions. Testing conditions and geometry of the specimens were in accordance with ASTM standards. Figure 2 shows the dimension of the prepared tensile sample as per ASTM standards, and all dimensions are in mm. The Vickers microindentation device (Shimuadzu, HMV 2T E, Kyoto, Japan) was employed to measure the hardness with a 0.49 N load for 15 s on either side of the weld. Tensile tests were carried out at room temperature on a universal testing machine (KAPPA 100 SS-CF, ZwickRoell, Fürstenfeld, Austria) with a strain rate of 0.0005 s^−1^. For each condition of the weld, three tests were conducted.

## 3. Results and Discussion

### 3.1. Microstructure: Base/Parent Metal

**Figure 3:**
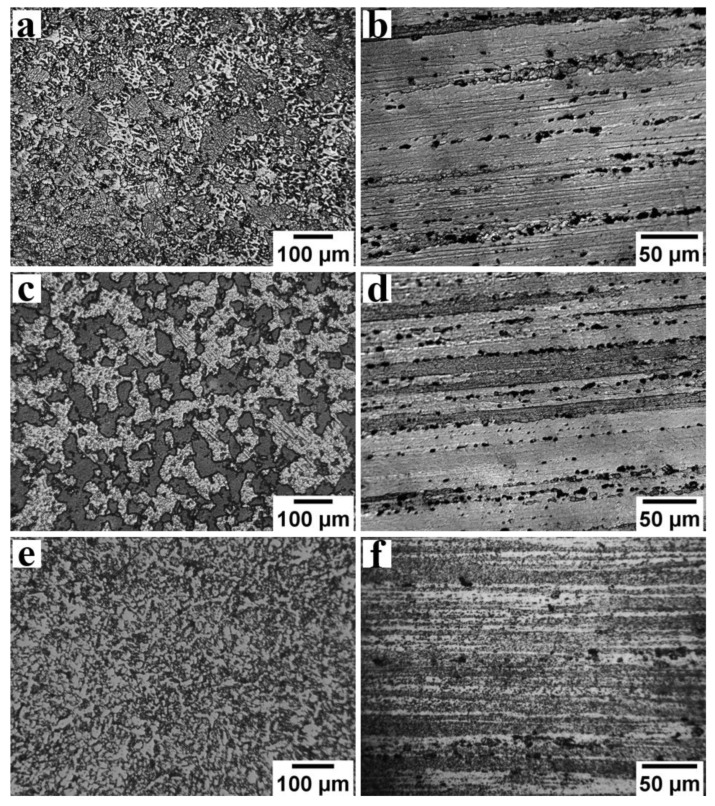
Optical micrographs along the transverse and longitudinal to the axis of the base alloy rods in AA7075-STA (**a**,**b**), AA7075-ST (**c**,**d**), and AA5083 (**e**,**f**).

**Figure 4:**
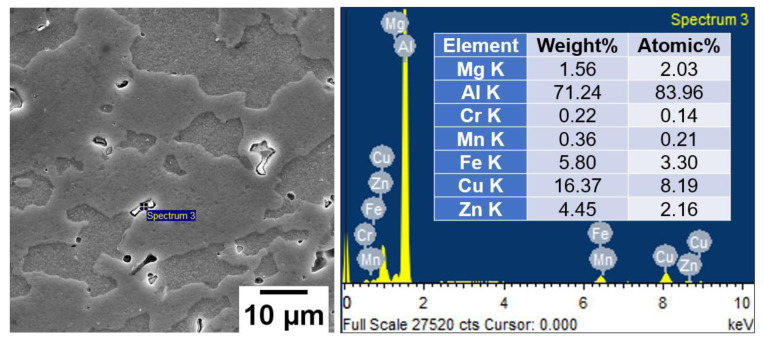
SEM image and EDS analysis of the AA7075-ST (Base).

Figure 3 features the optical micrographs taken on a plane transverse (Figure 3a,c,e) and longitudinal (Figure 3b,d,f) to the axis of the base alloy rods in all heat treatment conditions. The optical microstructure of AA7075-STA samples showed elongated pancake-shaped grains with a length of 250 ± 8 µm along the longitudinal plane (Figure 3b), while the transverse plane showed grains with equiaxed morphology (Figure 3a) with an average diameter of 80 ± 7 µm. Similar grain morphologies can be observed in AA7075-ST and AA5083 alloys, as shown in Figure 3c–f. The average grain diameter of AA5083 is 91 ± 5 µm (longitudinal plane) with a grain length (transverse plane) of 200 ± 9 µm. The observed grain structures, viz., the pancake-shaped grains along the rod axis, are the result of rolling operation during the manufacturing of these rods. Furthermore, scanning electron micrographs (Figure 4) of the AA7075-ST sample show grain morphologies and second-phase particles present in the microstructure. AA7075 is an age-hardened alloy; the main constituent of the strengthening of this alloy is the metastable η′ (MgZn2) precipitate, which is formed during aging [21,22]. A typical strengthening of AA7075 starts with the α-phase followed by the formation of GP (Guinier–Preston) zones–metastable MgZn2 (η′) phase–Stable MgZn2 (η) phase [23,24]. The superior strength is achieved by the strong resistance of the pinning effect by these precipitates to dislocation [25]. The EDS pattern and the composition of the second phase particle are shown in Figure 4. It was found that the second phase particles are rich in Cu and Fe and identified as the Al_7_Cu_2_Fe phase [26]. Zou et al. [27] observed that this Al_7_Cu_2_Fe phase is stable at temperatures as high as 480 °C because of the higher melting temperature of this phase. Hence, in the current study, the solution treatment, which was carried out at a temperature of 468 °C for 2 h, could not dissolve the Al_7_Cu_2_Fe phase. Moreover, the differential etching of the adjacent grains could be seen, which resulted in the smooth and rough grain surface morphologies.

**Figure 5:**
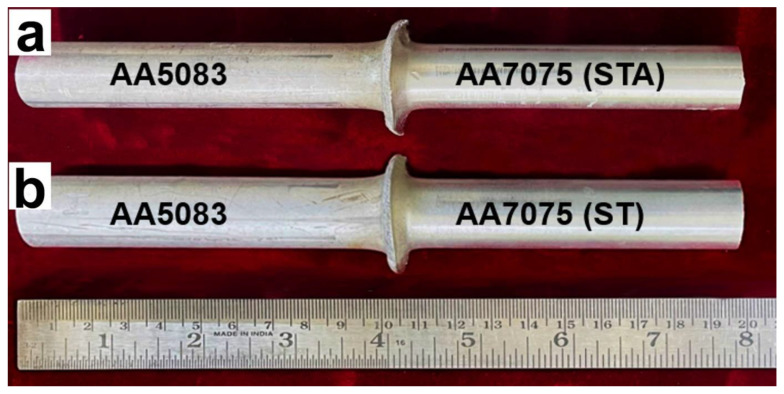
Visual view of welded joints (**a**) AA5083/AA7075-STA and (**b**) AA5083/AA7075-ST.

**Figure 6:**
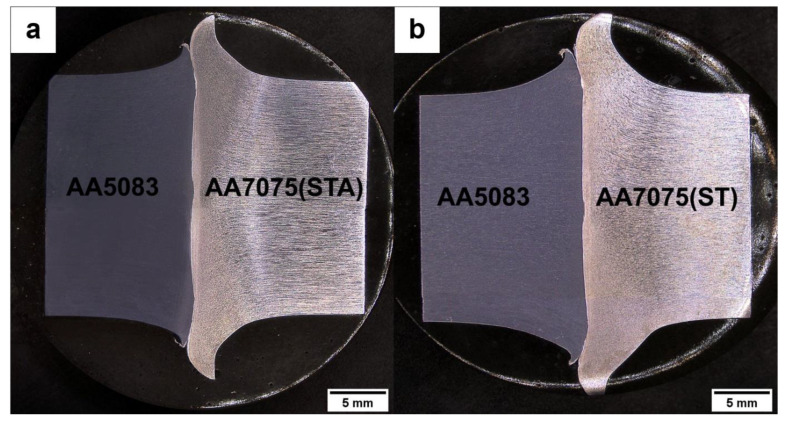
Macrographs of the weldment (**a**) AA5083/AA7075-STA and (**b**) AA5083/AA7075-ST.

The rotary friction welded joints (AA5083/AA7075-ST and AA5083/AA7075-STA) are shown in Figure 5. Good quality joints are observed, as uniform flash is seen without any snag; this reveals that enough frictional heat was produced during welding. For all weld conditions, lopsided flash was produced along the weld interface with higher flash (i.e., more deformation) formed on the AA7075 side compared to AA5083 (Figure 6). This is because the melting temperature of AA7075 (around 477 °C) is lower than that of AA5083 (approximately 591 °C) [28,29]. The lower melting point of AA7075 results in higher softening at welding temperatures, causing a higher flash compared to the AA5083 alloy.

### 3.2. Microstructure: Weldment

**Figure 7:**
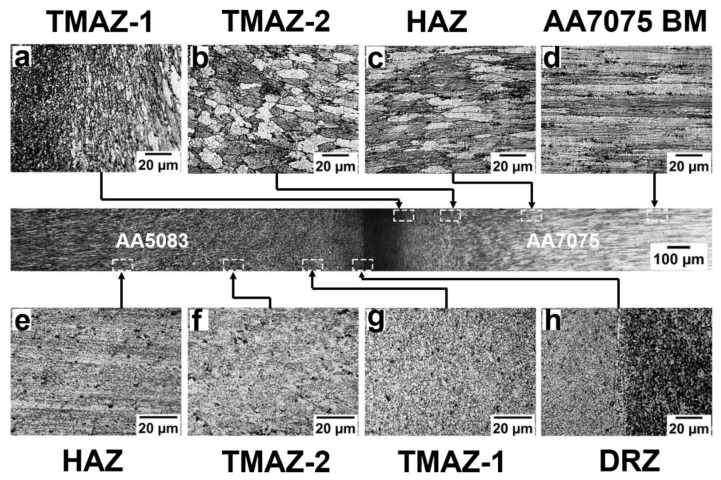
Microstructure across the weld interface of AA5083/AA7075-STA. AA7075 side: (**a**) TMAZ-1, (**b**) TMAZ-2, (**c**) HAZ, (**d**) base metal; AA5083 side: (**e**) HAZ, (**f**) TMAZ-2, (**g**) TMAZ-1, (**h**) DRZ.

**Figure 8:**
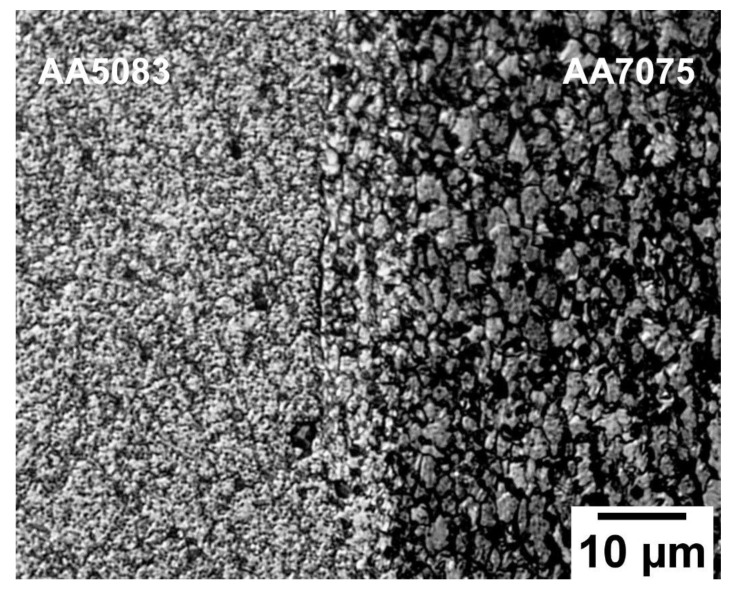
Optical microstructure at the DRZ of AA5083/AA7075-STA welds.

Figure 7 depicts the grain structure variation at different zones of rotary friction weldment AA5083/AA7075-STA. The microstructural examination identified several welding zones, including the (i) dynamically recrystallized zone (DRZ)/weld zone, (ii) thermomechanically affected zone (TMAZ), (iii) heat affected zone (HAZ), and (iv) base metal (BM) on both sides of the weld interface [5]. These zones are common for all heat-treated conditions (i.e., ST, STA, and PWHT). The DRZ of AA5083/AA7075-STA consists of very fine grains (as shown in Figure 7h) on both sides of the weld interface. The dynamic recrystallization during welding resulted in fine equiaxed grains with an average grain size of 4 ± 2 μm on the AA7075 side of the STA weldment. This is due to the combined effect of high temperature and severe plastic deformation occurring in this zone. Figure 8 depicts a magnified image of the DRZ with the fine equiaxed grains produced due to the severe recrystallization caused during welding. Jata et al. [30] studied the recrystallization mechanism of friction stir welded Al-Li-Cu plates and found that the grain boundary migration and recrystallized nuclei formation are due to the continuous dynamic recrystallization (CDRX) process. This process involves a gradual relative rotation of adjacent grains through dislocations glide [30]. Further, the differential state of stress and metal flow occurring during the various stages (friction stage and forging stage) of friction welding led to variation in the grain structure of the TMAZ, which is characterized into (i) TMAZ-1 and (ii) TMAZ-2. Compared to the DRZ, lower/partial recrystallization is seen in TMAZ. Figure 7a shows the grain structure of TMAZ-1, which is adjacent to the DRZ on the AA7075 side. The grains in TMAZ-1 are elongated and parallel to the weld interface, resulting from plastic deformation during the frictional stage of the welding. The average grain size (21 ± 6 μm) is slightly higher in this zone compared to the DRZ. TMAZ-2 contains almost circular equiaxed grains resulting from upset pressure during the forging stage of friction welding. The HAZ displays pancake-shaped coarse grains with an increase in grain thickness along the line perpendicular to the longitudinal axis of the grains. The average grain size value of the HAZ is found to be 50 ± 8 μm. Similar variations in the grain morphology can be seen on the AA5083 side. Fine equiaxed grains can be observed in the DRZ of AA5083 with a grain size of 5 ± 2 μm. Highly deformed grains structure in the TMAZ and coarse grain structures in the HAZ are observed at the AA5083 side of the AA5083/AA7075-STA weldment, as shown in Figure 7e–g.

**Figure 9:**
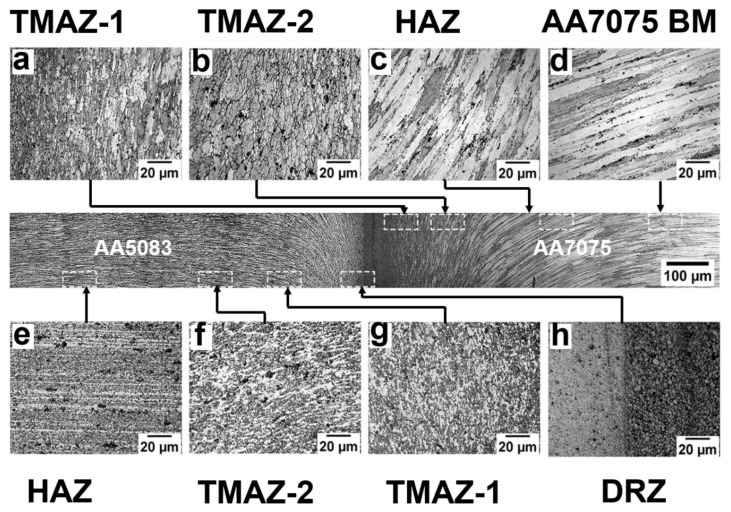
Microstructure across the weld interface of AA5083/AA7075-ST. AA7075 side: (**a**) TMAZ-1, (**b**) TMAZ-2, (**c**) HAZ, (**d**) base metal; AA5083 side: (**e**) HAZ, (**f**) TMAZ-2, (**g**) TMAZ-1, (**h**) DRZ.

The weld zones fairly similar to the STA weld condition are also evident in the AA5083/AA7075-ST weldment, as shown in Figure 9. Fine equiaxed (recrystallized) grains can be seen in the DRZ on the AA7075 side of the weldment with an average grain size of 5 ± 2 μm. Deformation flow lines can be observed along the TMAZ (Figure 9) due to severe plastic deformation and metal flow occurring during welding. The HAZ displayed a recrystallized grain structure that can be attributed to the thermal cycle during the welding. The AA5083 side of the weldment also displayed a similar variation in the microstructures across the zones due to the thermal cycle and plastic flow during the welding.

**Figure 10:**
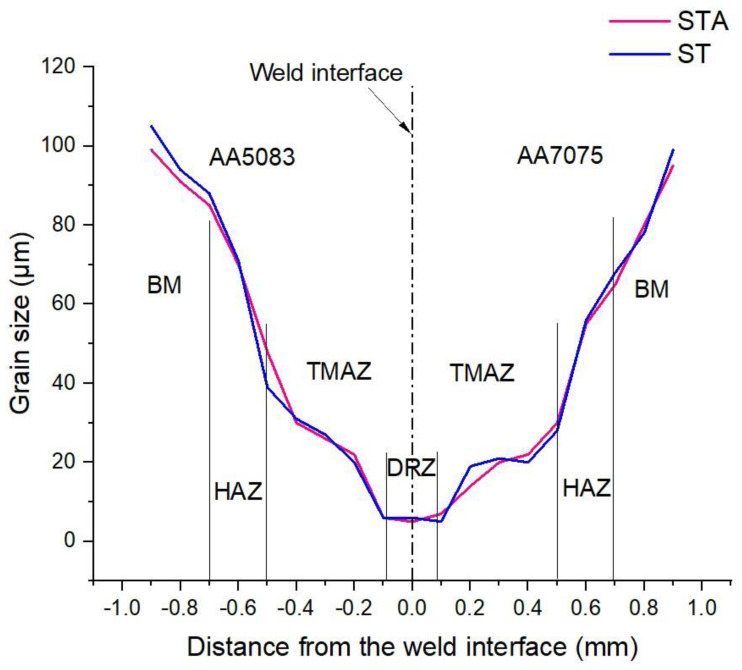
Average grain size variation across different weld zones of AA5083/AA7075 welds.

Figure 10 displays the average grain sizes across various weld zones of AA5083/AA7075 ST and STA welds. The results indicate that the DRZ has the smallest average grain size compared to the TMAZ, HAZ, and base metal on either side. This could be due to the occurrence of dynamic recrystallization during friction welding. It is important to note that average grain sizes are the same in different weld zones of AA5083/AA7075 ST and STA conditions. In the AA5083/AA7075 weldment, the average grain size of the DRZ is 5 ± 2 µm on the AA5083 side and 4 ± 2 µm on the AA7075 side, while the TMAZ has an average grain size of 31 ± 9 µm on the AA5083 side and 21 ± 6 µm on the AA7075 side. On both sides of the AA5083/AA7075 weldment, the HAZ is observed adjacent to the TMAZ. Since the HAZ is relatively far from the weld interface, it experiences heat and less plastic deformation, resulting in no recrystallization. As a result, the grains that form in the HAZ are coarser than those in the TMAZ and FDZ zones. The average grain size values of the HAZs are 67 ± 9 µm and 50 ± 8 µm on the AA5083 and AA7075 sides, respectively. Notably, significant grain coarsening is observed in the HAZ of the AA5083 side, which may be attributed to the annealing effect during welding, resulting in the loss of work hardening compared to the HAZ of the AA7075 side. Finally, the BM is observed on both sides of the AA5083/AA7075 weldment adjacent to the HAZ.

**Figure 11:**
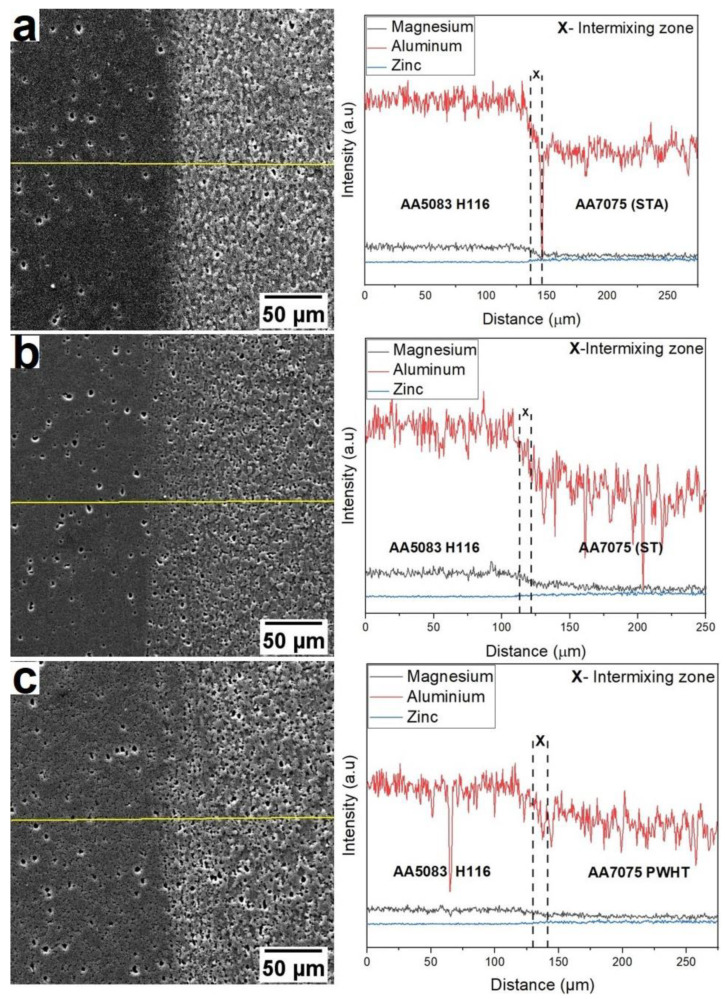
EDS (line scan) microanalysis: elemental distribution across weld interface (**a**) AA5083/AA7075-STA, (**b**) AA5083/AA7075-ST, and (**c**) AA5083/AA7075-PWHT.

Figure 11 shows the SEM micrographs of the weld interface along with the EDS line scan. The existence of the intermixed zone can be seen with the help of an EDS line scan. A similar intermixing zone at the weld interface of the IN718/IN600 dissimilar friction weld was reported by Rehman et al. [31]. The metal mixing at the weld interface is due to the severe mechanical deformation and the rods’ relative motion during welding. The major alloying elements of either metal, i.e., magnesium (for AA5083) and zinc (for AA7075), are seen to blend in the intermixing zone as observed from the EDS line scans. The formation of the intermixing zone is common for weldments prepared under all the given heat treatment conditions (viz., STA, ST, and PWHT).

### 3.3. Mechanical Properties

**Figure 12:**
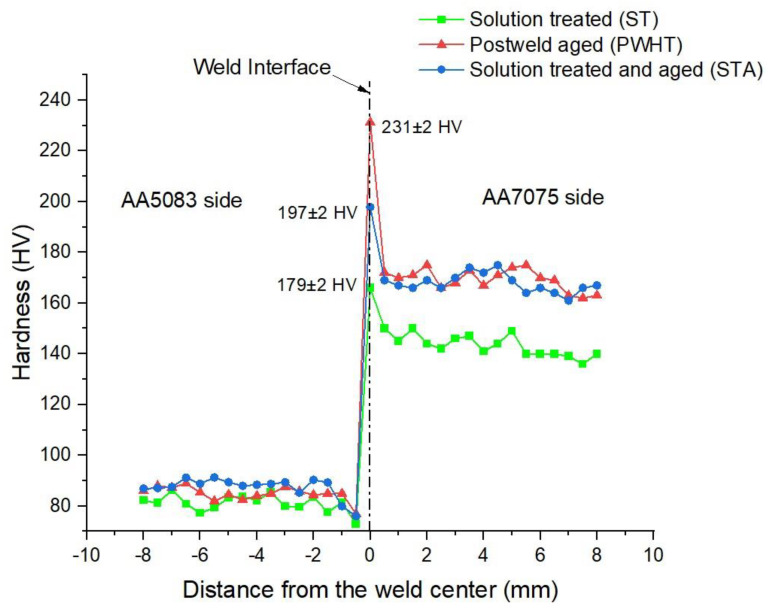
Hardness distribution across the weld interface for all heat treatment conditions.

Figure 12 shows the hardness distribution across the weldment for all three heat treatment conditions. The DRZ exhibits the maximum hardness for each weld condition (ST, STA, and PWHT), mainly due to the severe recrystallization that takes place during welding, leading to grain refining and producing finer grains [4]. Therefore, it can be stated that the heat produced during welding is sufficient for recrystallization. The highest hardness (231 ± 2 HV) at the weld interface can be seen in AA5083/AA7075-PWHT along the DRZ region. This could be attributed to the combined effect of grain refinement and the formation of strengthening secondary phases (such as MgZn_2_) during postweld aging [12]. Additionally, when compared to PWHT weld condition, AA5083/AA7075-STA displayed lower hardness at the DRZ (197 ± 2 HV). This can be attributed to the partial dissolution of these secondary/strengthening phases (such as MgZn_2_) at welding temperature along the weld interface. Xu et al. [32] confirmed that only 5 min is enough to dissolve MgZn_2_ precipitates entirely at 475 °C. The overall lowest hardness (179 ± 3 HV) is observed in AA5083/AA7075-ST. This could be attributed to the dissolution of major phases and forming a supersaturated solid solution during solution treatment [25]. Moreover, the lowest hardness (173 ± 2 HV) along the AA5083 side was seen at 0.5 mm (HAZ) from the weld interface. This decrease in hardness value was therefore found to be caused by the presence of coarser grains in the HAZ. Due to the differential nature of the strengthening mechanism and metallurgical properties of the respective alloys AA5083 and AA7075, an asymmetrical hardness distribution graph can be seen across the weldment.

**Figure 13:**
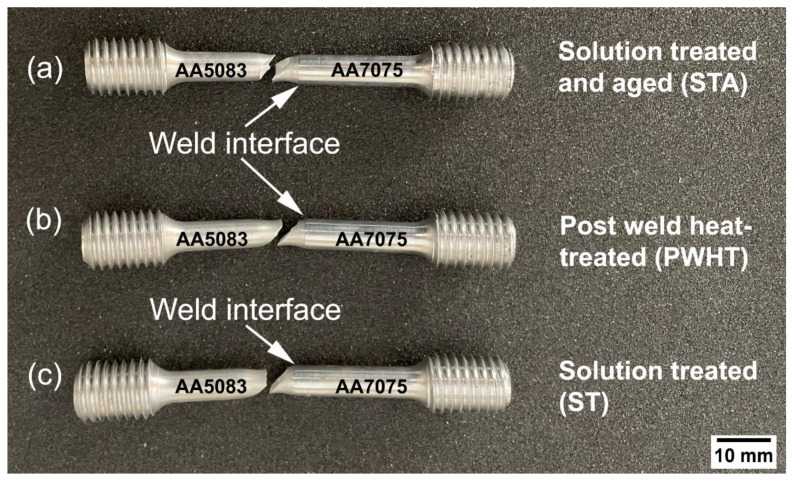
Showing tensile failure of (**a**) AA5083/AA7075-STA, (**b**) AA5083/AA7075-PWHT, and (**c**) AA5083/AA7075-ST.

Figure 13 shows the fractured samples for the various heat-treated weld conditions during tensile tests. As shown in the figures, it is clear that the failure occurred on the AA5083 HAZ side of the weldment. The superior mechanical properties of the AA7075 base (yield strength (YS): 600 ± 6 MPa, ultimate tensile strength (UTS): 642 ± 6 MPa) compared to the AA5083 base (YS: 196 ± 3 MPa, UTS: 340 ± 2 MPa) controlled the crack initiation at AA5083. In dissimilar welding, a rule of thumb for qualification of the weld is that the failure should occur in the weaker of the two base materials away from the weld [33]. In all heat treatment conditions, failure occurred at the HAZ of the AA5083 side where the lowest hardness levels were recorded (Figure 12) due to the annealing effect during welding, causing loss of work hardening and grain coarsening in the HAZ [34]. Therefore, due to the coarser grains present in the HAZ compared to TMAZ, the fracture occurred at the HAZ.

Therefore, it can be stated that the strength of the weldment (at the weld interface) is higher than AA5083 for every heat treatment condition (STA, ST, and PWHT). Table 4 shows the detailed tensile properties of the weldments as well as the base metals in various heat treatment conditions. All heat treatment conditions displayed a ductile-type failure with visible necking and failed surfaces angled at 45° to the axis of the shaft.

**Figure 14:**
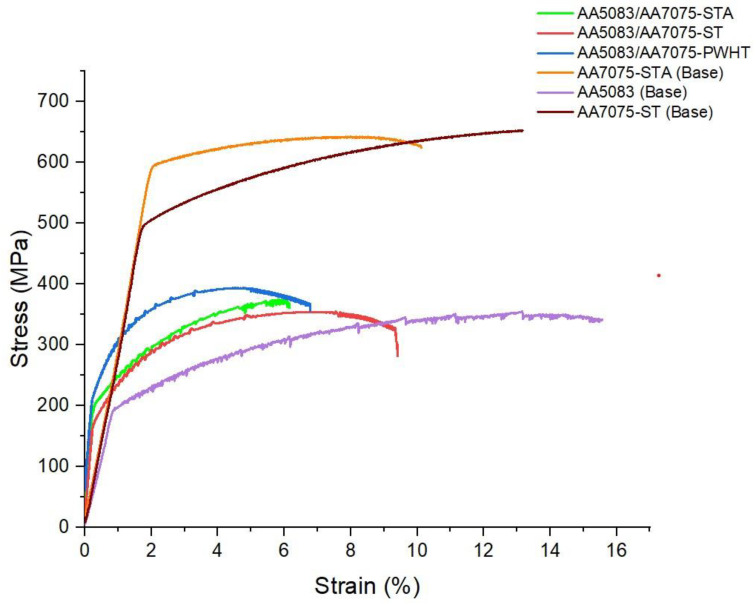
Tensile curves of welded samples and base metals.

Figure 14 shows the typical tensile curves of the welded samples (STA, ST, and PWHT condition) along with the base metal. The rotary friction welded sample AA5083/AA7075-PWHT joint displays the highest strength (YS:195 ± 3 MPa, UTS:387 ± 2 MPa) and low ductility (6 ± 1% elongation) among all the other welded conditions. The superior tensile properties of AA5083/AA7075-PWHT can be correlated to the presence of the major strengthening precipitates MgZn_2_ (of AA7075) formed during postweld aging, as they act as a catalyst in strengthening the weldment [12]. On comparing STA and ST weld conditions, the partial dissolution of these precipitates during welding resulted in higher strength and low ductility of AA5083/AA7075-STA welds (YS: 190 ± 2 MPa, UTS: 360 ± 4 MPa and % elongation: 6 ± 1) compared to AA5083/AA7075-ST welds (YS: 178 ± 6 MPa, UTS: 345 ± 5 MPa and % elongation: 9 ± 2). These changes can be related to the absence of secondary phases (such as MgZn_2_, etc.) in ST conditions, which also increase the ductile nature of the weld [32]. Furthermore, AA5083/AA7075-STA displayed a lower strength (YS: 190 ± 2 MPa, UTS: 360 ± 4 MPa) compared to the AA5083/AA7075-PWHT. This can be attributed to the partial dissolution of the secondary phases due to the thermal exposure during welding at the weld interface [35].

The AA5083 H116 base metal sample displays a comparatively lower strength (YS: 196 ± 3 MPa, UTS: 340 ± 2 MPa) with a % elongation of 15 ± 2 compared to AA7075 STA and AA7075 ST base metal samples. Serrated flow is observed in the σ–ε curve of AA5083 base samples; further investigation revealed that it is due to the phenomenon known as the Portevin–Le Chatelier (PL) effect, which is quite common for Al-Mg alloys [36]. The dynamic interaction between the solute atoms and mobile dislocations leads to this effect. These mobile dislocations migrate between numerous obstacles in a series of starts and stops. At these obstructions, solute atoms are momentarily stopped before diffusing around the dislocations, thus leading to negative strain sensitivity [37]. The σ–ε curves of all the welded samples display these serrated flow/yielding since the samples failed on the AA5083 side for all heat treatment conditions.

**Figure 15:**
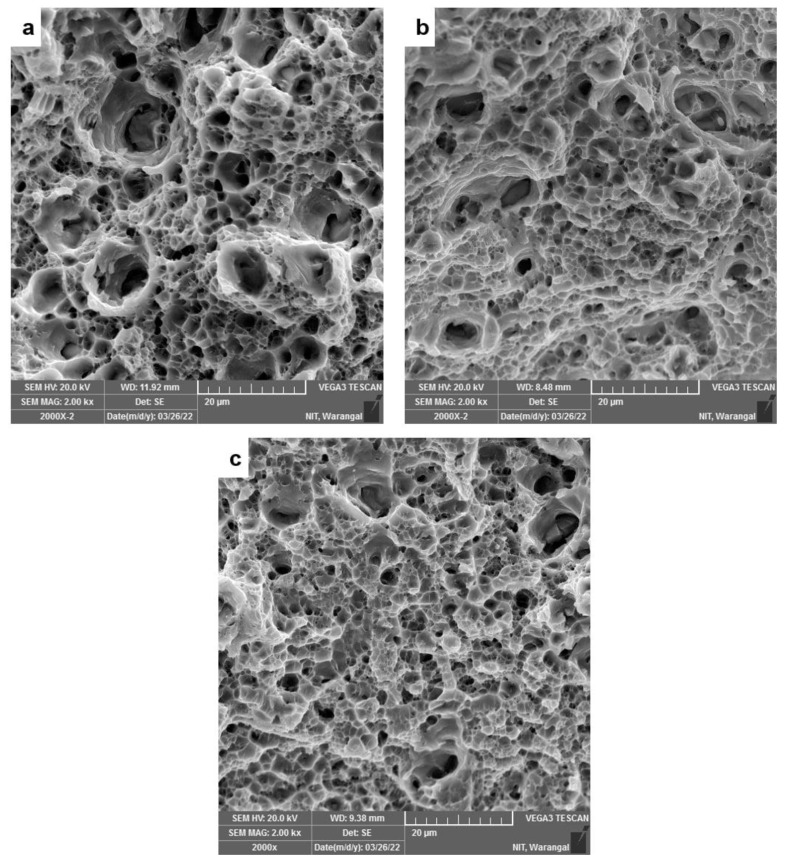
Fractographs of rotary friction welded samples (**a**) AA5083/AA7075-STA, (**b**) AA5083/AA7075-ST, and (**c**) AA5083/AA7075-PWHT.

The contribution of microstructural characteristics to the joint’s local strength and ductility can be determined by examining the tensile fracture surface [10]. A ductile type of failure can be verified with the SEM micrographs of the fracture surface for all samples. Figure 15 displays the fractography of the failed surface (AA5083 side) for the respective heat treatment conditions of STA, ST, and PWHT. With higher magnification, the morphologies reveal a variety of voids of different sizes and forms that are dispersed throughout the fracture surface. The fracture occurred on the AA5083 side of the weldment. As a result, a similar failure pattern can be observed for all heat-treated conditions of STA, ST, and PWHT.

## 4. Conclusions

In the current study, the dissimilar AA5083/AA7075 welds were prepared using the rotary friction welding technique. The investigation focused on how the macrostructure and microstructure impacted the hardness and tensile properties of both the as-welded (ST and STA conditions) and PWHT conditions of the dissimilar AA5083/AA7075 welds. The summary of conclusions is as follows:

1. Defect-free dissimilar AA5083/AA7075 welds, such as cracks or porosity, could be obtained with a rotary friction welding technique.
2. The macrostructure of dissimilar AA5083/AA7075 welds displayed more flash on the AA7075 side, and little flash on the AA5083 side, and this can be attributed to AA7075 having a lower melting point and higher softening at friction welding temperatures.
3. Significant grain refinement was observed at the weld interface (DRZ) due to the continuous dynamic recrystallization occurring during rotary friction welding. The region next to DRZ experienced lower strain and high temperatures, resulting in the formation of deformed grains in the TMAZ.
4. The rotary friction welded sample AA5083/AA7075-PWHT joint exhibited the highest strength (YS: 195 ± 3 MPa, UTS: 387 ± 2 MPa) among all the other welded conditions, and this may be attributed to the major strengthening precipitates MgZn_2_ (of AA7075) formed during postweld aging. On the contrary, the partial dissolution of these precipitates during welding resulted in higher strength and low ductility of AA5083/AA7075-STA welds (YS: 190 ± 2 MPa, UTS: 360 ± 4 MPa and % elongation: 6 ± 1) compared to AA5083/AA7075-ST welds (YS: 178 ± 6 MPa, UTS: 345 ± 5 MPa and % elongation: 9 ± 2).
5. All dissimilar welds failed in the HAZ region of the AA5083 side due to the formation of coarse grains, indicating the weakest region.

## Figures and Tables

**Table 1:** Chemical composition of base metals.

Materials/Weight%	Zn	Fe	Cu	Cr	Mg	Si	Mn	Al
AA7075 T6511	5.92	0.15	1.93	0.193	2.8	0.05	0.01	Bal
AA5083 H116	0.15	0.31	0.042	0.086	4.76	0.18	0.60	Bal

**Table 2:** Heat treatment details.

Material	Heat Treatment
AA7075 (Base)	Solutioning treatment (at 468 °C for 2 h)-[ST]
	Solutioning (at 468 °C for 2 h) and artificial aging (at 120 °C for 24 h)-[STA]
AA7075 (ST)-AA5083 [Weld]	Postweld aging of the joint (120 °C for 24 h)

**Table 3:** Parameter employed for welding.

Parameters	Investigated Range	Finalized
Rotational speed (rev/min)	1200–1400	1400
Friction burn-off (mm)	2–5	5
Upset force (kN)	19–24	24
Soft force (kN)	(Constant)	2
Soft force time (sec)	(Constant)	4
Friction force (kN)	(Constant)	12
Upset time (sec)	(Constant)	3

**Table 4:** Results of tensile tests of base metals as well as the weldments.

Tensile Properties			
Condition	Yield Stress (YS), MPa	Ultimate Tensile Strength (UTS), MPa	Percentage Elongation (%)
AA7075 T651 STA (Base)	600 ± 6	642 ± 6	10 ± 1
AA7075 ST (Base)	492 ± 7	649 ± 6	13 ± 3
AA5083 H116 (Base)	196 ± 3	340 ± 2	15 ± 2
AA5083/AA7075-STA	190 ± 2	360 ± 4	6 ± 1
AA5083/AA7075-ST	178 ± 6	345 ± 5	9 ± 2
AA5083/AA7075-PWHT	195 ± 3	387 ± 2	6 ± 1

## Data Availability

Data are contained within this article.
